# *Helicobacter pylori* bacteria alter the p53 stress response *via* Erk-HDM2 pathway

**DOI:** 10.18632/oncotarget.2828

**Published:** 2015-01-22

**Authors:** Vikas Bhardwaj, Jennifer M. Noto, Jinxiong Wei, Claudia Andl, Wael El-Rifai, Richard M. Peek, Alexander I. Zaika

**Affiliations:** ^1^ Department of Veterans Affairs, Tennessee Valley Healthcare System, Nashville, Tennessee, USA; ^2^ Department of Surgery, Vanderbilt University Medical Center and Vanderbilt-Ingram Cancer Center, Nashville, Tennessee, USA; ^3^ Department of Cancer Biology, Vanderbilt University Medical Center and Vanderbilt-Ingram Cancer Center, Nashville, Tennessee, USA; ^4^ Division of Gastroenterology, Vanderbilt University Medical Center and Vanderbilt-Ingram Cancer Center, Nashville, Tennessee, USA

**Keywords:** p53, *Helicobacter pylori*, gastric tumor, HDM2, CagA

## Abstract

*H. pylori* infection is the strongest known risk factor for gastric cancer. Inhibition of host tumor suppressor mechanisms by the bacteria underlies the development of this disease. Among the tumor suppressors affected by *H. pylori* are p53 and E-cadherin, which inhibition has been shown to increase the risk of gastric cancer. In this report, we investigated the interaction between E-cadherin and p53 in *H. pylori*-infected cells. We found that downregulation of E-cadherin leads to cellular stress and activation of p53. In the setting of *H. pylori* infection, this mechanism, however, is disrupted. We found that although co-culture of gastric epithelial cells with *H. pylori* led to downregulation of E-cadherin and cellular stress, it resulted in inhibition of p53, which is mediated by intracellular Erk kinases and HDM2 protein induced by *H. pylori*. Experimental inhibition of HDM2/p53 interactions restored p53 activity, and decreased survival of infected cells. Collectively, our results revealed that regulation of p53 and E-cadherin is tightly linked through the p53 stress response mechanism that is inhibited by *H. pylori* via activation of Erk1/2-HDM2-p53 pathway leading to survival of damaged cells. This might be advantageous to the bacteria but may increase the cancer risk.

## INTRODUCTION

Approximately half of the world's population is infected with the bacterial pathogen *Helicobacter pylori* (*H. pylori*). Gastric colonization by *H. pylori* is the major cause of chronic gastritis and peptic ulcer disease, and is the strongest known risk factor for gastric adenocarcinoma (GC), which remains one of the most common forms of cancer and one of the leading causes of cancer-related death worldwide [1]. Interplay between host and bacterial factors plays a defining role in the development of gastric malignancy [2]. One of the best characterized bacterial virulence determinants known to increase the risk of gastric cancer is the *cag* pathogenicity island (*cag*PAI) [3]. The *cag*PAI is a bacterial genetic locus that encodes the type IV secretion system (T4SS), which is required for delivery of bacterial protein CagA into host cells. Following intracellular injection through the T4SS, CagA protein is phosphorylated by host kinases and alters the cell signaling machinery in ways that are thought to be beneficial to the bacteria. CagA was found to behave as a bacterial oncoprotein inducing gastric tumor in mice when it was transgenically overexpressed [4]. It has been shown that multiple oncogenic pathways such as PI3K/Akt, Wnt/β-catenin and Ras/Erk are activated by CagA and other bacterial factors.

The aberrant activation of these oncogenes is counteracted by cellular tumor suppressors such as p53. p53 protein is accumulated in stress conditions and activates transcription of multiple p53 targets that halt abnormal proliferation of cells that otherwise may become tumorigenic [5, 6]. The p53 gene (*tp53*) is the most commonly mutated gene in gastric tumor. Approximately 40–50% of gastric cancer patients carry inactivating mutations in the p53 gene. *H. pylori* infection has been reported to enhance mutagenesis of p53 [7]. However, recent studies have also shown that *H. pylori* can cause non-mutational inactivation of p53 [8, 9].

*H. pylori* has been shown to preferentially attach to gastric epithelial cells in close proximity to the apical junctional complexes [10]. Bacterial attachment has been suggested to disrupt the epithelial barrier and possibly provide the bacteria with essential nutrients or access to the lamina propria [11]. Disruption of the epithelial barrier involves alteration of the components of the junctional complexes including E-cadherin [12]. E-cadherin is a component of the adherens junctions that regulates cell-cell adhesion, intracellular signaling, cell polarity, and proliferation [10, 13]. *H. pylori* infection has shown to acutely cleave E-cadherin and disrupt the adherens junctions [14, 15]. *H. pylori* infection can also decrease expression of E-cadherin by promoting hypermethylation of the E-cadherin gene (*CDH1*) that can be reversed by eradication of the bacteria [16–18]. The E-cadherin gene (*CDH1*) was found to be frequently mutated in diffuse type of gastric tumor, implying that E-cadherin inactivation is important for gastric cancer development [19]. However, animal studies have found that downregulation of E-cadherin alone is not sufficient to cause gastric tumor. In addition to E-cadherin, inactivation of p53 is required for development of gastric malignancy in mice [20]. E-cadherin and p53 mutations have also been shown to increase predisposition to gastric cancer in human [21, 22]. In this study, we investigated the interaction between p53 and E-cadherin in *H. pylori*-infected cells.

## RESULTS

### *H. pylori* decreases E-cadherin protein levels

**Figure 1:**
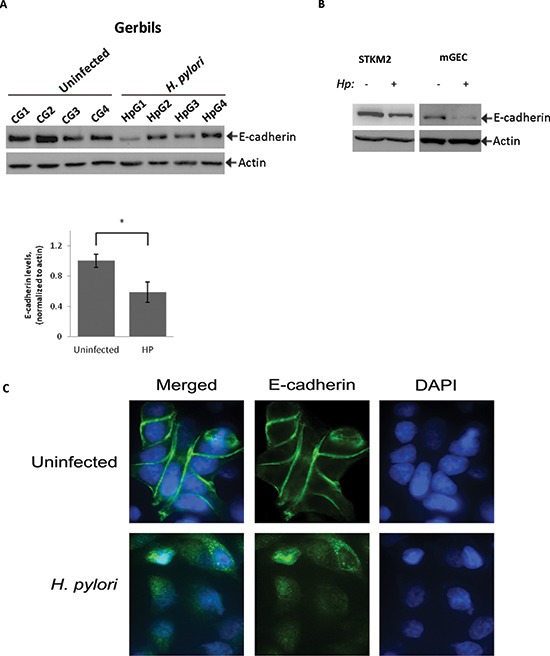
*H. pylori* downregulates E-cadherin protein *in vitro* and *in vivo* **(A)** Lysates were prepared from gastric tissues collected from Mongolian gerbils infected with *H. pylori* strain 7.13 for 6 hrs or left uninfected and analyzed using Western blotting. Actin was used as a loading control. Protein levels of E-cadherin were quantified by densitometry and compared between uninfected (CG1…CG4) and *H. pylori*-infected (HpG1…HpG4) groups of animals (*, *p* < 0.05). **(B)** E-cadherin protein was analyzed in STKM2 and mGEC cells co-cultured with *H. pylori* strain 7.13 for 24 hrs. **(C)** Immunofluorescence analysis of expression and subcellular localization of E-cadherin protein was performed in STKM2 cells co-cultured with *H. pylori* strain 7.13 for 24 hrs. A representative image is shown.

As a prelude to defining the role of p53-E-cadherin interactions, we initially sought to characterize the regulation of E-cadherin in Mongolian gerbils infected with *H. pylori*. Mongolian gerbils were inoculated with *H. pylori* strain 7.13 for 6 hours and then gastric tissues were collected and analyzed for E-cadherin expression by Western blotting. We found that E-cadherin protein levels were significantly decreased following infection of Mongolian gerbil with *H. pylori* (Figure 1A). To determine the effects of *H. pylori* on gastric epithelial cells in a more controlled environment, we first analyzed expression of E-cadherin protein in a panel of gastric epithelial cells harboring wild-type p53 (AGS, HFE145, SNU1, STKM2 and mGEC). Detectable levels of E-cadherin were only observed in STKM2 and mGEC cells (Supplementary Figure 1A). STKM2 cells and mGECs were then co-cultured with *H. pylori* strain 7.13 at a bacteria-to-cell ratio of 100:1 for 24 hours and analyzed for alterations in E-cadherin protein levels. We found that *H. pylori* strongly reduces the levels of E-cadherin in both STKM2 and mGEC cells (Figure 1B). Since mGECs express SV40 large T-antigen that may affect regulation of p53 [23], we used STKM2 cells for our further analysis.

We next performed immunofluorescence analysis of E-cadherin protein in STKM2 cells co-cultuted with *H. pylori* strain 7.13 (MOI 100) for 24 hours and found that *H. pylori* disrupts intercellular adherens junctions and reduces protein levels of E-cadherin, as judged by a decrease in intensity of fluorescence (Figure 1C). Combined, our results show that *H. pylori* acutely downregulates E-cadherin levels and disrupts cellular adherens junctions in our model system.

### Inhibition of E-cadherin activates p53

**Figure 2:**
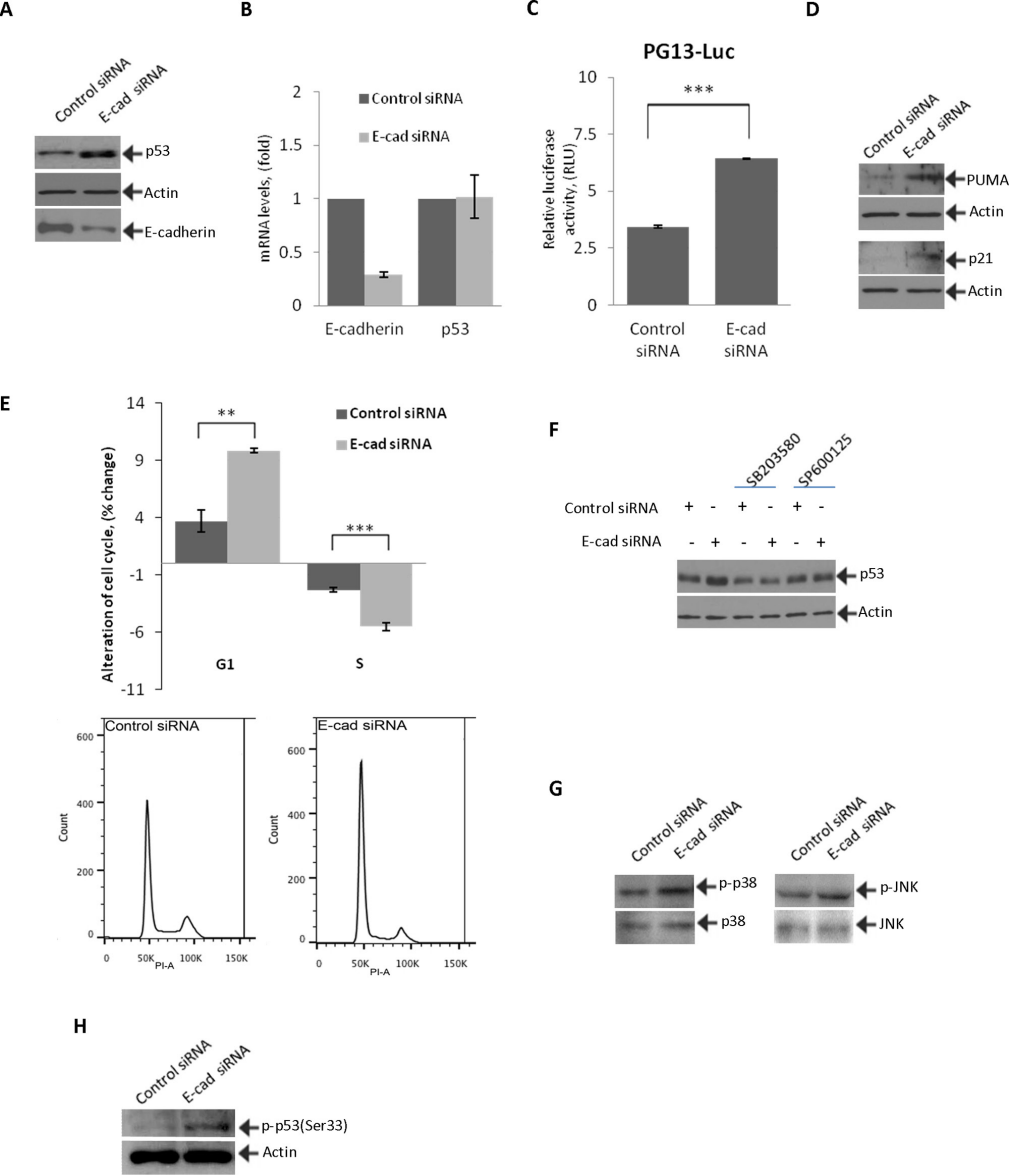
Inhibition of E-cadherin activates p53 and induces cell cycle arrest **(A)** STKM2 cells were transfected with either specific E-cadherin or control siRNA for 48 hrs and analyzed for expression of p53 protein by Western blotting. **(B)** STKM2 cells transfected with either control siRNA or E-cadherin siRNA for 48 hrs were analyzed for mRNA expression of E-cadherin and p53. Data were normalized to expression of HPRT mRNA. **(C)** STKM2 cells treated with a scrambled control siRNA or E-cadherin siRNA for 24 hrs were co-transfected with p53 reporter plasmid (PG13-Luc) and vector expressing Renilla luciferase for an additional 24 hrs and analyzed using the dual-luciferase reporter assay (***, *p* < 0.001; *n* = 3). **(D)** The same as (A) but protein expression of p53 transcriptional targets, p21 and PUMA, was assessed. **(E)** Cell cycle analysis was performed on STKM2 cells transfected with E-cadherin or control scrambled siRNA for 48 hrs by flow cytometry using propidium iodide (PI) staining (**, *p* < 0.01. ***, *p* < 0.001; *n* = 4). Graph represents alterations in the G1 and S phases of the cell cycle (top panel). Bottom panels show flow cytometry profiles for STKM2 cells transfected with either control or E-cadherin siRNAs. **(F)** Inhibition of p38 and JNK kinases prevents induction of p53. STKM2 cells transfected with control or E-cadherin siRNAs for 36 hrs were treated with specific inhibitors (5 μM) of p38 (SB203580) or JNK (SP600125) kinases for an additional 12 hrs and analyzed for expression of p53 protein using Western blotting. **(G)** Phosphorylation of p38(Thr180/Tyr182) and JNK(Thr183/Tyr185) was assessed after downregulation of E-cadherin with siRNA. **(H)** Phosphorylation of p53 protein at Ser33 was analyzed after downregulation of E-cadherin in STKM2 cells.

To investigate how downregulation of E-cadherin affects p53, STKM2 cells were transfected with E-cadherin siRNA and analyzed for expression of p53. p53 protein was found to be strongly induced in response to E-cadherin downregulation (Figure 2A). Notably, this induction of p53 was not accompanied by changes in levels of p53 mRNA, indicating that p53 protein is regulated by post-translational mechanisms (Figure 2B). To analyze how downregulation of E-cadherin affects activity of p53, we employed a reporter assay. STKM2 cells treated with control or E-cadherin siRNAs for 24 hours were transfected with p53 luciferase reporter PG13-Luc and analyzed for p53 activity. We found that inhibition of E-cadherin significantly activates the p53 reporter (Figure 2C). Downregulation of E-cadherin was also accompanied by an increase in protein levels of p53 transcriptional targets PUMA and p21/WAF1, further showing that p53 becomes activated (Figure 2D). To analyze the functional role of p53 activation, we performed cell cycle analysis after E-cadherin downregulation using flow cytometry. We found that inhibition of E-cadherin leads to cellular stress and G1/S cell cycle arrest (Figure 2E) but does not result in apoptosis (Supplementary Figure 1B).

To investigate the mechanism of p53 induction, STKM2 cells transfected with E-cadherin siRNA were treated with specific chemical inhibitors that affect upstream regulators of p53. Protein lysates were collected 12 hours after treatment and analyzed for expression of p53 protein using Western blotting. Among the tested compounds, p38 MAPK (SB203580) and JNK/SAPK (SP600125) inhibitors were most effective in suppression of p53 (Figure 2F and data not shown). These stress kinases are known to directly phosphorylate and activate p53 [24, 25]. To assess the kinase regulation, their phosphorylation was analyzed after downregulation of E-cadherin with siRNA. An increased phosphorylation of both p38(Thr180/Tyr182) and JNK(Thr183/Tyr185) kinases was found, indicating their activation (Figure 2G). We also found an increased phosphorylation of p53 at position serine-33 (Ser33), a phosphorylation site regulated by p38 kinase [26], implying that E-cadherin downregulation leads to activation of stress kinases and p53 (Figure 2H).

### *H. pylori* prevents activation of p53 and decreases its protein levels

**Figure 3:**
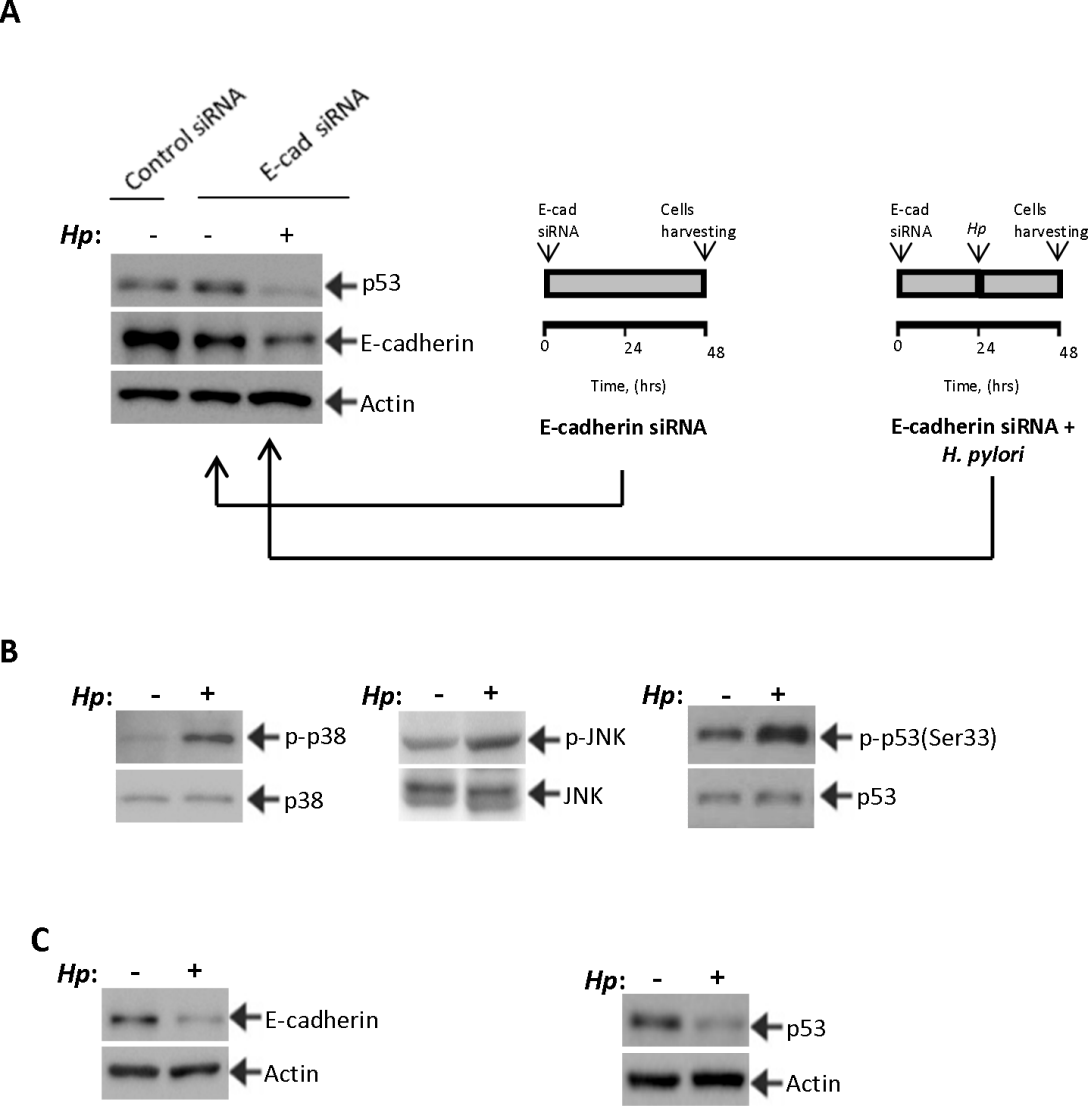
*H. pylori* inhibits p53 induction **(A)** *H. pylori* inhibits the p53 response. STKM2 cells treated with E-cadherin siRNA for 24 hrs were co-cultured with *H. pylori* strain 7.13 for an additional 24 hrs and analyzed for expression of p53 (left panel). The right panels illustrate the experimental design. **(B)** *H. pylori* activates p38 MAPK and JNK stress kinases. The effect of *H. pylori* strain 7.13 on phosphorylation of p38 and JNK proteins was assessed in STKM2 cells. An increased phosphorylation of p38(Tyr180/Tyr182) and JNK1(Thr183/Tyr185) was found 1.5 hrs and 24 hrs after co-culture with *H. pylori*, respectively. Phosphorylation of p53 protein at serine 33 was also increased. Since *H. pylori* decreases protein levels of p53, expression of p53(Ser33) was normalized to total p53 protein levels. **(C)** Co-culture with *H. pylori* led to downregulation of E-cadherin and p53 proteins.

To investigate the effect of *H. pylori* on activation of p53, STKM2 cells in which E-cadherin was downregulated with siRNA for 24 hours were co-cultured with *H. pylori* strain 7.13 (MOI 100) for an additional 24 hours. We found that *H. pylori* completely prevents induction of p53 and further decreases E-cadherin protein levels, implying that *H. pylori* is capable of inhibiting the p53 response in cells in which E-cadherin was downregulated (Figure 3A). To investigate the mechanism of p53 regulation in *H. pylori*-infected cells, we first analyzed the regulation of stress kinases p38 MAPK(Thr180/Tyr182) and JNK1(Thr183/Tyr185) in STKM2 cells co-cultured with *H. pylori* strain 7.13. Analogous to experiments shown in Figure 2, we found an increase in phosphorylation of p38 and JNK1 kinases as well as p53 protein at serine 33 (Figure 3B). We also found downregulation of E-cadherin (Figure 3C). However, despite downregulation of E-cadherin and activation of the stress kinases, levels of p53 protein were decreased, showing that *H. pylori* can prevent p53 response in cells with E-cadherin downregulation (Figure 3C).

### p53 and E-cadherin are regulated in an HDM2-dependent manner

**Figure 4:**
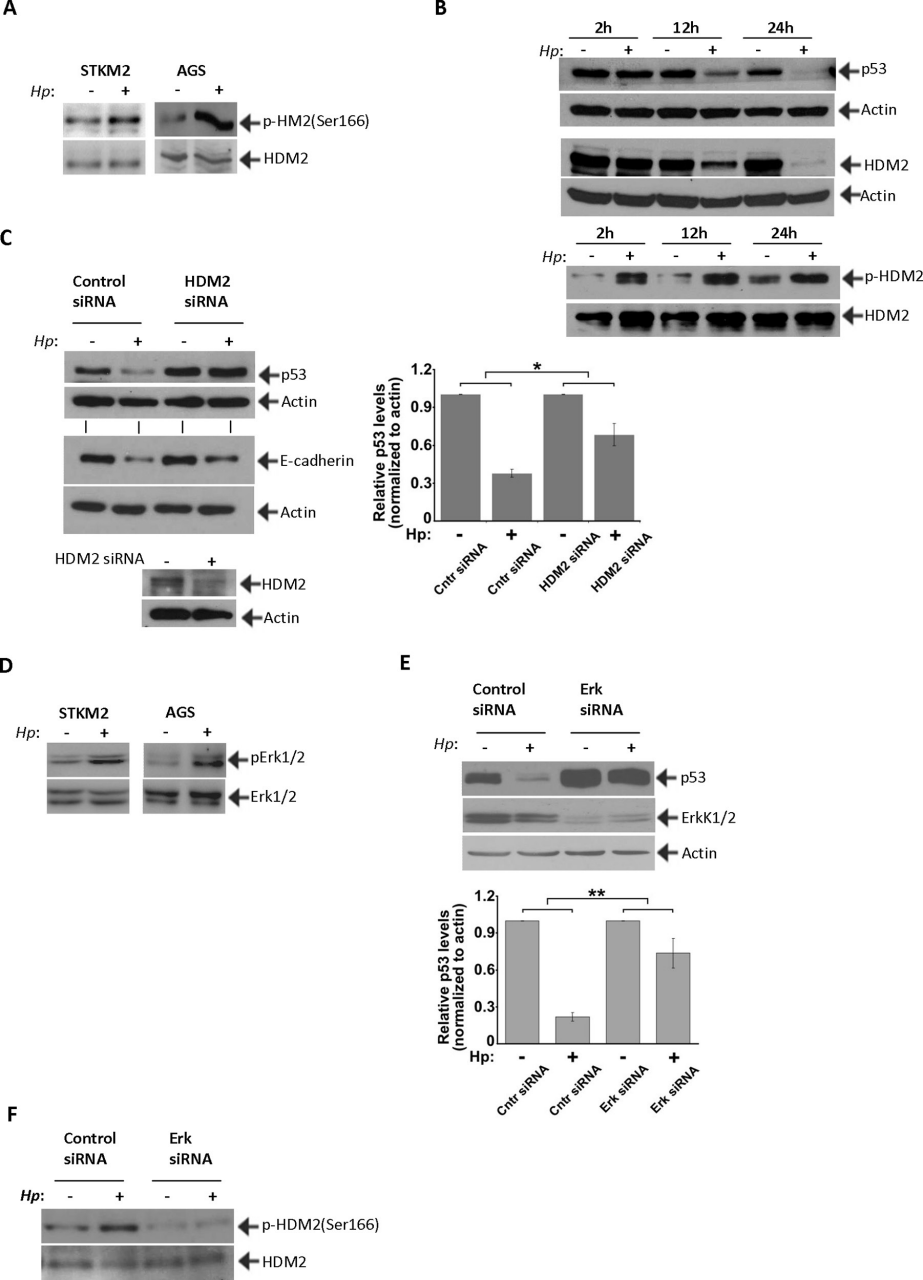
HDM2 regulate p53 in *H. pylori*-infected cells **(A)** *H. pylori* increases phosphorylation of HDM2 protein. Effect of *H. pylori* on phosphorylation of HDM2(Ser166) was analyzed in STKM2 and AGS cells co-cultured with *H. pylori* strain 7.13 for 8 hrs. Phosphorylation of HDM2 was normalized to total HDM2 protein levels. **(B)** Protein levels of HDM2 are dynamically changed in *H. pylori*-infected cells and follow kinetics that are similar to those of p53. At the same time, relative phosphorylation levels of HDM2(Ser166) protein are strongly increased. **(C)** Downregulation of HDM2 with specific siRNA inhibits degradation of p53 protein induced by *H. pylori* (*p* < 0.05; mean ± SEM; *n* = 3). Expression of p53 protein was quantified by densitometry, normalized to actin and arbitrarily set at 1 in uninfected cells. STKM2 cells were transfected with HDM2 siRNA for 24 hrs followed by co-culture with *H. pylori* strain 7.13 for additional 24 hrs and then analyzed for expression of p53 and E-cadherin proteins. Bottom panel shows inhibition of HDM2 protein by siRNA. **(D)** The effect of *H. pylori* on phosphorylation of Erk1/2(Tyr204) was analyzed in STKM2 and AGS cells co-cultured with *H. pylori* strain 7.13 for 4 hrs. Levels of phosphorylated Erk1/2 kinases were normalized to total levels of Erk1/2 proteins. **(E)** Downregulation of Erk1/2 kinases leads to significant inhibition of p53 degradation (*p* < .01; mean ± SEM; *n* = 4) by *H. pylori*. AGS cells treated with Erk1/2 siRNA for 24 hrs were co-cultured with *H. pylori* strain J166 for an additional 24 hrs and analyzed for protein expression of p53 and Erk1/2 by Western blotting. Expression of p53 protein was quantified by densitometry and normalized to actin expression (bottom panel). Expression of p53 in control uninfected cells was arbitrarily set at 1. **(F)** The effect of Erk1/2 inhibition on protein phosphorylation of HDM2(Ser166) in AGS cells co-cultured with *H. pylori* strain J166 was assessed using Western blotting. Phosphorylation of HDM2 was normalized to total HDM2 protein levels.

Given that E3 ubiquitin ligase HDM2 is a critical regulator of p53 protein, we next investigated regulation of HDM2 in *H. pylori*-infected cells. We found that co-culture of STKM2 cells with *H. pylori* strain 7.13 increases the phosphorylation of HDM2 at Ser166 (Figure 4A) which is known to increase the activity of HDM2 [27, 28]. An increase in activation of HDM2 was also found in AGS cells co-cultured with *H. pylori* (Figure 4A). Since AGS cells express low levels of E-cadherin (Figure Supplemental 1A), it suggests that activation of HDM2 by *H. pylori* is independent of E-cadherin expression. Interestingly, protein levels of HDM2 are dynamically decreased in *H. pylori*-infected cells and follow the degradation kinetics of p53 (Figure 4B). At the same time, a relative phosphorylation level of HDM2(Ser166) protein is steadily increased (Figure 4B, bottom panel).

To determine the role of HDM2 in *H. pylori*-infected cells, STKM2 cells were transfected with HDM2 specific siRNA and co-cultured with *H pylori*. Inhibition of HDM2 significantly suppressed the downregulation of p53 protein (*p* < .05) induced by *H. pylori* (Figure 4C). We then sought to investigate the mechanisms that regulate HDM2 in *H. pylori-*infected cells. A previous report has shown that activation of Erk(Tyr204) increases phosphorylation of HDM2 at serine 166 [29]. We investigated the effect of *H. pylori* on Erk1/2 activation and found that *H. pylori* increases the phosphorylation of Erk2(Tyr204) in STKM2 and AGS cells (Figure 4D). We next inhibited Erk1/2 by specific siRNA for 24 hours followed by co-culture with *H. pylori* for an additional 24 hours. Our results show that downregulation of Erk1/2 significantly inhibits degradation of p53 (*p* < .01) induced by *H. pylori* (Figure 4E) and activates transcriptional targets p53, p21 and PUMA (Supplementary Figure 1C). Inhibition of Erk1/2 also reduces phosphorylation of HDM2(Ser166) showing that activation of Erk kinases by *H. pylori* activates HDM2, which in turn, inhibits p53 (Figure 4F).

### Bacterial CagA protein is involved in the regulation of p53

**Figure 5:**
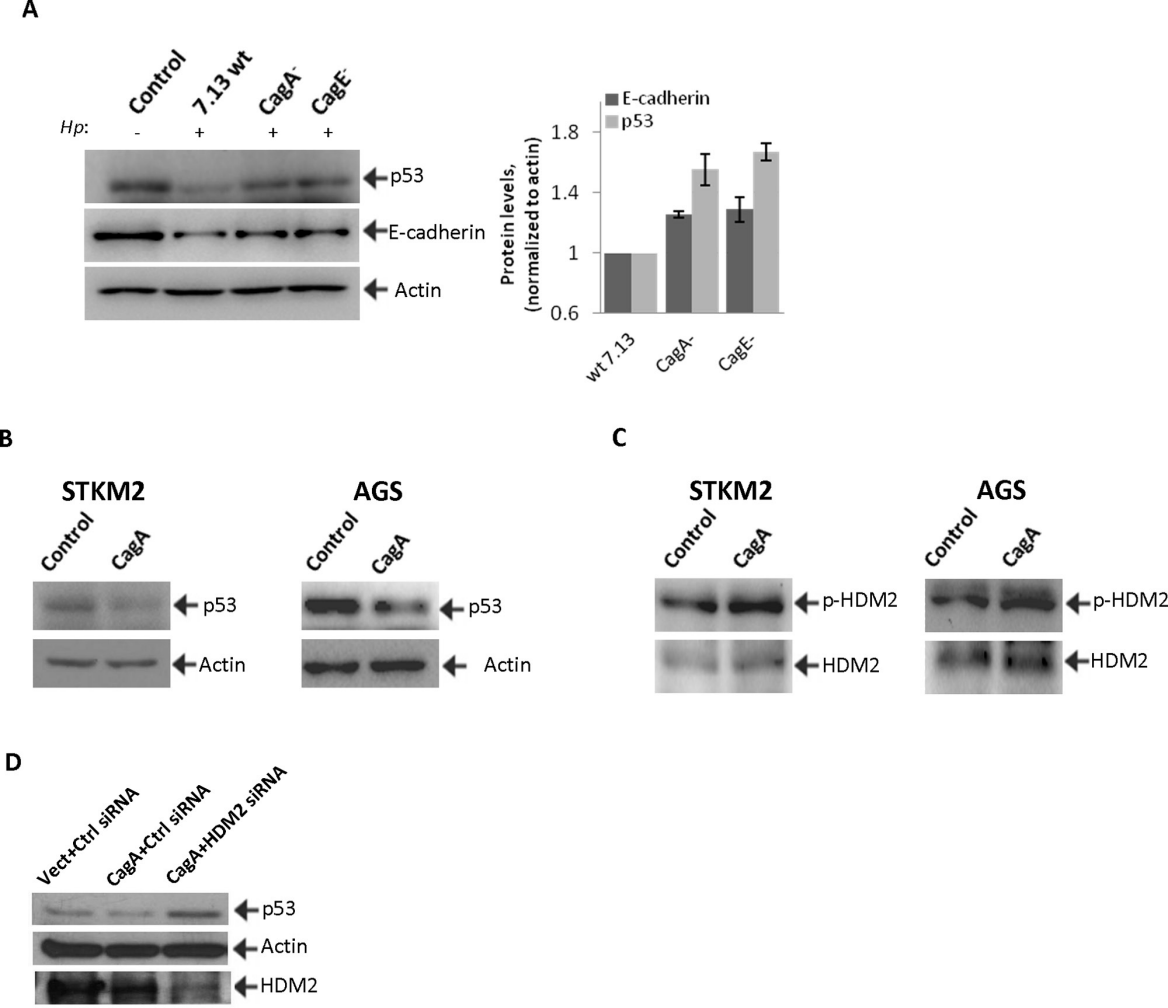
CagA is involved in the regulation of HDM2 and p53 proteins in *H. pylori*-infected cells **(A)** Cell lysates were collected from STKM2 cells co-cultured with wild type *H. pylori* strain 7.13 or its isogenic mutants *cag*A^−^ and *cag*E^−^ for 24 hrs and analyzed for protein expression of p53 and E-cadherin. Right panel shows densitometric quantifications of p53 and E-cadherin proteins. **(B)** STKM2 and AGS cells were transfected with CagA-expressing plasmid, cagA-pSP65SRα, for 24 hrs and analyzed for expression of p53 protein. **(C)** The same as (B) but phosphorylation of HDM2 protein was analyzed. **(D)** STKM2 cells were co-transfected with the indicated plasmids (empty vector or CagA) and either scrambled siRNA or HDM2 siRNA for 40 hrs and then analyzed for expression of p53 and HDM2 proteins. Bottom panel shows inhibition of HDM2 protein by siRNA.

Given that injection of bacterial CagA protein plays an important role in the alteration of multiple intracellular signaling pathways, we next assessed the role of CagA in regulation of p53 and E-cadherin. We co-cultured STKM2 cells with wild-type *H. pylori* strain 7.13 and its isogenic *cag*A^−^ and *cag*E^−^ mutants for 24 hours (MOI 100) and analyzed for expression of p53 and E-cadherin proteins. The latter mutant is functionally similar to 7.13 *cagA*^−^ as it does not form the T4SS that is required for delivery of CagA into host cells. The strongest inhibition of p53 was found by wild-type bacteria, while the mutants were less potent. E-cadherin protein was downregulated by all bacterial strains, though significantly stronger inhibition was observed after co-culture with the wild-type bacteria (Figure 5A).

To further explore the role of CagA, we transfected STKM2 and AGS cells with CagA expression plasmid, cagA-pSP65SRα. We found that CagA transfection downregulates p53 protein level in both cell lines (Figure 5B). CagA transfection also increases phosphorylation of HDM2(Ser166) protein in STKM2 and AGS cells (Figure 5C). To investigate the role of HDM2 in the CagA signaling, STKM2 cells were co-transfected with CagA and HDM2 siRNA for 40 hours and analyzed for expression of p53 protein. We found that, while CagA inhibits p53, downregulation of HDM2 increases p53 protein levels in CagA-transfected cells, further supporting data that degradation of p53 by *H. pylori* is mediated by CagA and HDM2 (Figure 5D).

### Restoration of p53 activity increases cell death in *H. pylori*-infected cells

**Figure 6:**
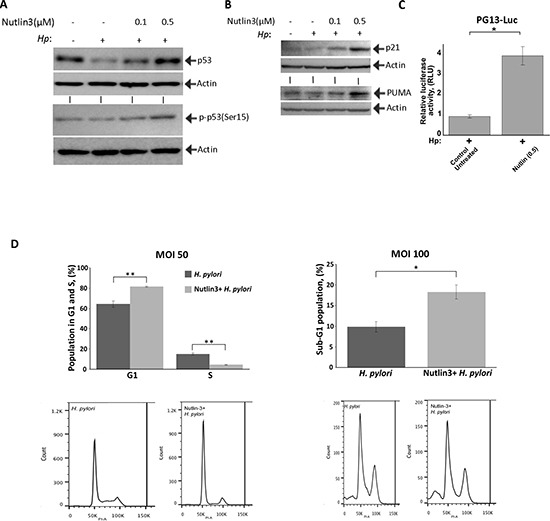
Nutlin-3 restores p53 activity and increases cell death of *H. pylori*-infected cells **(A)** Treatment with Nutlin-3 increases levels of p53 protein and its phosphorylation at Ser15. STKM2 cells treated with the indicated concentrations of Nutlin-3 were co-cultured with *H. pylori* strain 7.13 for 24 hrs and analyzed for expression of p53 protein (top) and its phosphorylation at Ser15 (bottom) by Western blotting. **(B)** The same as (A) but expression of p21 and PUMA proteins was analyzed. **(C)** STKM2 cells transfected with a p53 reporter plasmid (PG13-Luc) and treated with 0.5 μM Nutlin-3 were co-cultured with *H. pylori* strain 7.13 for 24 hrs and analyzed using the dual-luciferase reporter assay. **(D)** STKM2 cells treated with 5 μM Nutlin-3 or a vehicle were co-cultured with *H. pylori* strain 7.13 for 24 hrs at MOI 50 or MOI 100 and then collected, stained with propidium iodine, and analyzed by flow cytometry. Bar graph shows changes in the G1 and S phases of the cell cycle (left panel; **, *p* < 0.01; *n* = 3). Right panel shows the percentage of cells in the sub-G1 fraction (*, *p* < 0.05; *n* = 3). Bottom panels show flow cytometry profiles for the aforementioned experiments.

To analyze the biological role of p53 downregulation, we inhibited HDM2 with specific chemical inhibitor Nutlin-3, which hinders the interaction of p53 with HDM2. STKM2 treated with 0.1 μM or 0.5 μM Nutlin-3 were co-cultured with *H. pylori* strain 7.13 for 24 hours and analyzed by Western blotting. We found that Nutlin-3 restores p53 levels and increases phosphorylation of p53 protein at Ser15 in infected cells (Figure 6A). An increase in p53 protein levels was associated with an increase in p53-dependent transcription, since Nutlin-3 treatment enhanced p53 reporter activity (PG13-Luc) and upregulated p53 transcription targets p21 and PUMA (Figure 6B, 6C). Protein levels of HDM2 were also increased (Supplementary Figure 1D). To exclude a possibility that Nutlin-3 has a toxic effect on *H. pylori*, CagA delivery was assessed in Nutlin-3-treated cells. As a marker for delivery of CagA, we assessed tyrosine phosphorylation of CagA protein, since CagA is specifically phosphorylated by intracellular tyrosine kinases after its injection into gastric epithelial cell [30]. Our studies found that treatment with Nutlin-3 (0.5 μM) does not affect phosphorylation of CagA implying that Nutlin-3 does not have deleterious effects on *H. pylori* in these conditions (Supplementary Figure 1E). To assess how p53 restoration affects infected cells, we next performed the cell cycle analysis using flow cytometry. The effect of p53 restoration was evaluated at a MOI of 50 and MOI of 100. We found that treatment with Nutlin-3 increases G1/S cell cycle arrest in STKM2 cells co-cultured with *H. pylori* at a MOI of 50 (Figure 6D, left panel). At a higher bacteria-to-cell ratio (MOI 100), treatment with Nutlin-3 led to significant increase in cell death (Figure 6D, right panel) showing that inhibition of p53 by *H. pylori* leads to better survival of infected cells.

## DISCUSSION

Here we report that *H. pylori* can modulate the p53 response in stress conditions caused by targeted disruption of adherens junctions by *H. pylori* bacteria. We found that when E-cadherin is experimentally downregulated in gastric epithelial cells, it leads to strong cellular stress, activation of p53, induction of p53 transcription targets (p21/Waf1, PUMA), and G1/S cell cycle arrest. This process is mediated by induction of stress kinases p38 MAPK and JNK/SAPK, which have been previously reported to directly phosphorylate and activate p53 protein [24, 25]. In agreement with these data, we found an increased phosphorylation of p53 protein at Ser33 regulated by p38 kinase. We also found that inhibition of p38 and JNK kinases prevents induction of p53 in cells in which E-cadherin was downregulated. These findings are consistent with animal studies showing an increased expression of p53 and p21/Waf1 proteins in murine gastric lesions where the E-cadherin (*CDH1*) gene was conditionally deleted while concomitant knockdown of E-cadherin and p53 abrogates expression of p21, strongly increasing cell proliferation and gastric tumorigenesis in mice [20].

Studies conducted by us, as well as previous reports showed that *H. pylori* reduces E-cadherin protein levels and disrupts the adherens junctions [14, 15]. Exposure to *H. pylori* also induced p38 and JNK kinases. However, it does not lead to induction of p53. Our experiments (Figures 3 and 4) revealed that *H. pylori* prevents induction of p53 in gastric cells, in which E-cadherin was downregulated by increasing phosphorylation and activity of HDM2 protein. Inhibition of HDM2 with siRNA or Nutlin-3 suppresses *H. pylori* – induces degradation of p53. Testing of additional inhibitors of HDM2 such as stapled peptides may help to further dissect the mechanism by which *H. pylori* inhibits p53 [31].

Interestingly, protein levels of HDM2 protein follow the dynamics of p53 downregulation suggesting that a feed back loop regulates levels of p53 and HDM2 proteins in *H. pylori*-infected cells [32]. Additional studies are needed to define this mechanism. We found that Erk kinases are responsible for an increased phosphorylation of HDM2 since its inhibition reduces HDM2 phosphorylation and increases levels of p53 in *H. pylori*-infected cells. Previous studies have reported that Akt kinase can also phosphorylate HDM2 in *H. pylori*-infected cells [33], suggesting that both Erk and Akt kinases contribute to activation of HDM2 and downregulation of p53.

These studies support previous findings that p53 is regulated in a CagA-dependent manner [33]. Indeed, ectopic expression of CagA increases phosphorylation of HDM2 and degradation of p53. Downregulation of HDM2 inhibits activity of CagA directed toward p53. We also found that *H. pylori cagA*^−^ or *cagE*^−^ mutants are less potent in inhibition of p53 than that of wild-type. In regards to regulation of E-cadherin by *H. pylori*, both CagA-dependent and -independent mechanisms have been previously reported [34, 35]. In our studies, we observed that although CagA is not absolutely required, its presence does increase the downregulation of E-cadherin.

*H. pylori* is known to preferably adhere to epithelial cells in close proximity to the apical-junctional complexes and target them possibly to gain access to essential nutrients or obtain entry to the lamina propria [10, 11]. Disruption of epithelial barrier, however, is accompanied by significant alteration in cellular homeostasis and cellular stress, in which p53 is a key regulator of cell cycle and apoptosis. Inhibition of p53 may be advantageous for *H. pylori* as it reduces cell death and is associated with activation of the immune system, which recognizes and targets cells under cellular stress [36]. In support, we found that inhibition of HDM2 by Nutlin-3 in cells exposed to *H. pylori* restore p53 activity and significantly increase cell cycle arrest and cell death.

**Figure 7:**
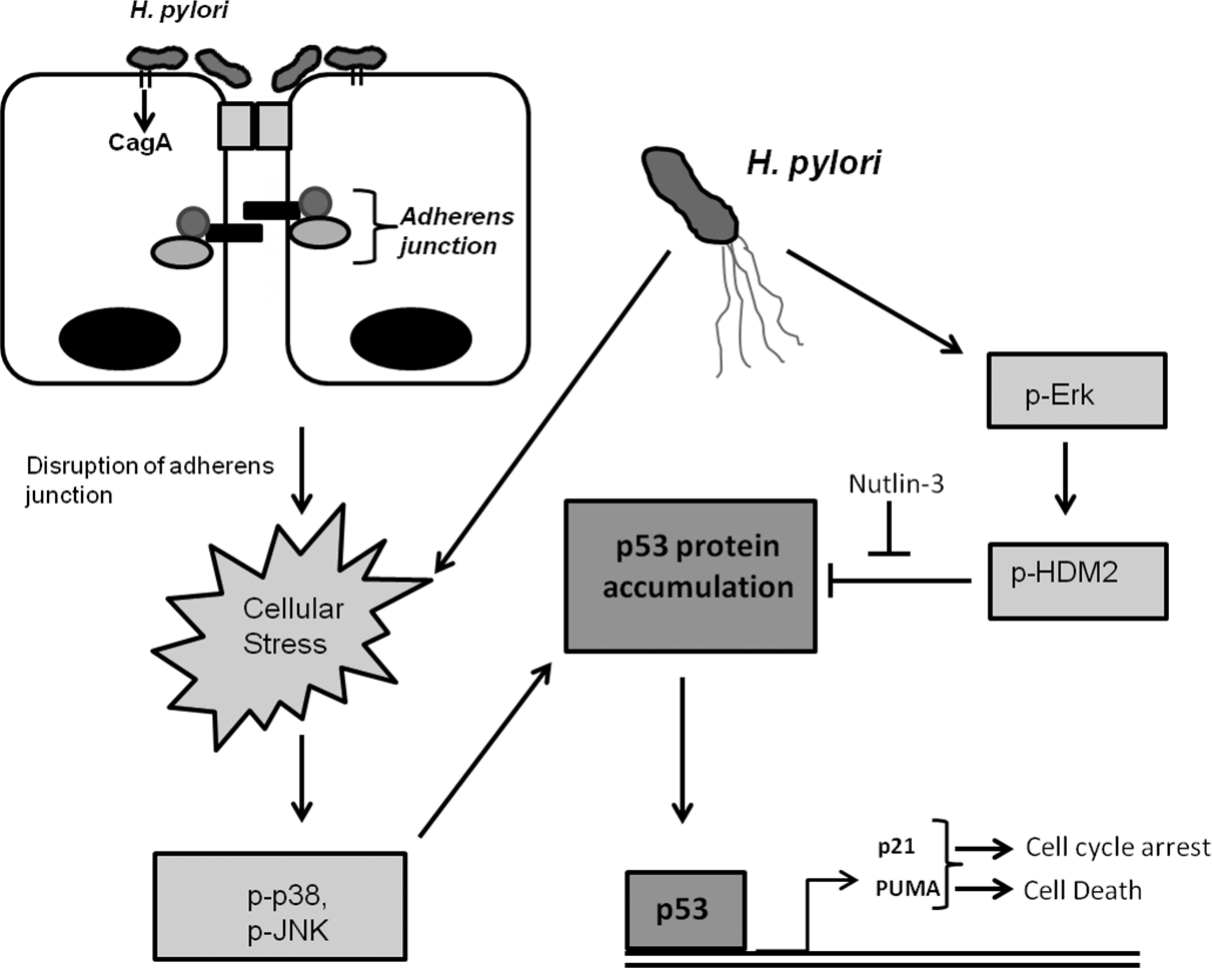
*H. pylori* alters p53 and E-cadherin signaling

Taken together, our studies show that regulation of E-cadherin and p53 is tightly linked through the p53 stress response mechanism. In the setting of *H. pylori* infection, this mechanism is inhibited as the bacteria modulate the p53 response through activation of the Erk1/2-HDM2 pathway that inhibits p53 in a CagA-dependent manner (Figure 7).

## MATERIALS AND METHODS

### Cell cultures and *H. pylori* infection

STKM2, AGS, SNU1, and HFE145 gastric cancer cells were maintained in RPMI 1640 medium (Invitrogen, Carlsbad, CA) supplemented with 10% fetal bovine serum (FBS). Murine gastric epithelial cells (mGECs) were harvested from a transgenic mouse, bearing a temperature-sensitive mutant of SV40 large T antigen and cultured in RPMI 1640 medium with 10% FBS and interferon-gamma at permissive temperature 33°C [23]. One day before experiment, mGEC cells were maintained at 37°C and continued until experimental end point.

CagA-positive *H. pylori* clinical strain J166 and rodent adapted strain 7.13 [37] were grown in *Brucella* broth with 5% FBS for 18 hours, harvested by centrifugation, and added to gastric cells at a bacteria-to-cell ratio of 100:1 (multiplicity of infection, MOI 100) unless otherwise mentioned. Isogenic *cag*A^−^ and *cag*E^−^ mutants were constructed within strain 7.13 by insertional mutagenesis using *aphA* and selection with kanamycin [37].

### Gerbil infection and western blotting

All animal experiments and procedures were approved by the Institutional Animal Care Committee at Vanderbilt University. Pathogen-free Mongolian gerbils purchased from Harlan Labs (Indianapolis, IN) were challenged orogastrically with either sterile Brucella broth or rodent-adapted *H. pylori* strain 7.13. The animals were euthanized 6 hour post infection; gastric tissues were harvested and analyzed by Western blotting.

### Luciferase reporter assay

PG13-Luc reporter plasmid has been described previously [38]. Luciferase activity was measured using the Dual Luciferase Reporter Assay Kit (Promega, Madison, WI) according to the manufacturer's protocol.

### Cell cycle and apoptosis analyses

Cell cycle analysis was performed as described previously [9]. Briefly, STKM2 cells were seeded into a 6-well plate and treated accordingly. The cells were trypsinized, collected, washed twice with PBS and fixed in 75% ethanol for 2 hours at 4°C. Cells were then washed twice with cold-PBS to remove ethanol and stained with propidium iodine (PI). The DNA content was measured by flow cytometry.

Apoptosis assay was performed using the Anexin V: PE Apoptosis Detection Kit I (BD Bioscience, San Jose, CA) according to the manufacturer's protocol.

### Plasmids, siRNA, antibodies, and chemical inhibitors

CagA expression plasmid (CagA-pSP65SRα) has been described previously [33]. E-cadherin siRNA and E-cadherin antibody were purchased from BD Bioscience. Antibodies to the following proteins were used in this study: p53 (DO-1), p21 (Ab-1), and HDM2 from Calbiochem (San Diego, CA); PUMA (ab9643) from Abcam Inc. (Cambridge, MA); p-MDM2(Ser166), JNK, p-p53(Ser33), p-p53(ser15), p-38, and p-p38(Thr180/Tyr182) from Cell Signaling Technology (Danvers MA); p-Erk1/2(Tyr204), p-JNK(Thr183/Tyr185) from Santa Cruz Biotechnologies (Dallas, TX). Protein loading was monitored using the anti–β-actin antibody (Sigma Aldrich, St. Louis, MO). Nutlin-3 was obtained from Sigma. Chemical inhibitor library was purchased from Selleckchem (Houston, TX). The following chemical inhibitors were used: p38 MAPK (SB203580), Akt(MK2206 2HCl), JNK/SAPK (SP600125), PKC delta (Go 6983), c-Met (PHA-665752), Rock-1 (Y-27632 2HCl), Rock-2 (Fasudil), Rock 1 and 2 (GSK429286A), FAK (PF-562271), PLK1 (Rigosertib), GSK3β (CHIR-98014), Src (Saracatinib), PKA (H89 2HCl), Fyn (PP1), DUBS (PR-619), mTOR1 and 2 (KU-0063794), STAT3 (WP1066), PI3K (LY294002), and Rac1 (EHop-016).

### RNA extraction and qRT-PCR

Total RNA was extracted using the Qiagen RNeasy kit. 1 μg RNA was reverse transcribed using the High-Capacity cDNA Reverse Transcription Kit according to the manufacturer's protocol (Applied Biosystem, Grand Island, NY). qRT-PCR was performed as described previously [9] with the following specific primers using the iCycler (Bio-Rad, Hercules, CA): E-cadherin: TGG AGG AAT TCT TGC TTT GC, CGT ACA TGT CAG CCA GCT TC; p53: TAA CAG TTC CTG CAT GGG CGGC, AGG ACA GGC ACA AAC ACG CAC C; HPRT: TTG GAA AGG GTG TTTA TTC CTC A, TCC AGC AGG TCA GCA AAG AA.

### Statistical analyses

Statistical analysis was performed using the Student's *t*-test. Results are expressed as averages ± SEM, if not specifically indicated. Results were considered significant when *p*-value was equal to or less than 0.05.

## SUPPLEMENTARY FIGURE



## Abbreviations

CagA: cytotoxin-associated gene A
MOI: Multiplicity of Infection
