# Supplementary material for: Helicobacter pylori bacteria alter the p53 stress response via Erk-HDM2 pathway
Source: Oncotarget. 2015 Jan 22;6(3):1531–43. doi: 10.18632/oncotarget.2828 (PMC4359312; doi:10.18632/oncotarget.2828)
Supplement: Supplementary file 1 [file oncotarget-06-1531-s001.pdf]

SUPPLEMENTARY FIGURE

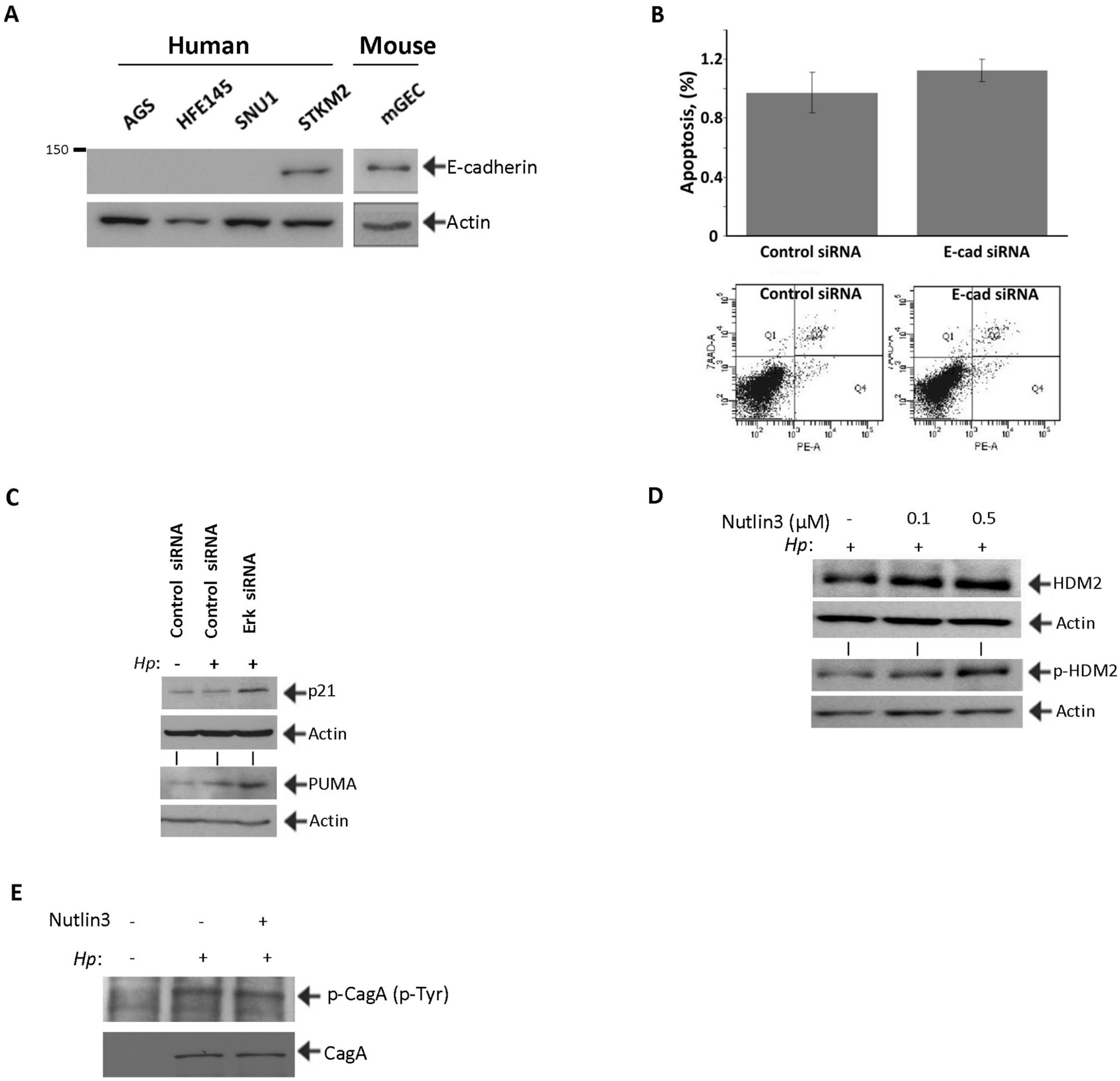

Supplementary Figure 1: (A) Western blot analysis of E-cadherin protein in gastric epithelial cell lines AGS, SNU1, HFE145 STKM2, and mGEC. **(B)** Analysis of cell death was performed on STKM2 cells transfected with E-cadherin or control scrambled siRNA for 48 hrs by flow cytometry using the Annexin V staining. **(C)** Expression of p53 target genes (p21 and PUMA) was assessed after downregulation of Erk kinases with siRNA (48 hrs) in infected AGS cells. **(D)** Treatment with Nutlin-3 increases levels and phosphorylation of HDM2 protein at Ser166. STKM2 cells treated with the indicated concentrations of Nutlin-3 were co-cultured with *H. pylori* for 24 hrs and analyzed for expression of HDM2 protein (top) and its phosphorylation at Ser166 (bottom) by Western blotting. **(E)** Nutlin-3 does not affect delivery of CagA protein into host cells. Phosphorylation of CagA protein was used as a marker for its delivery into host cells. STKM2 cells treated with 0.5 μM Nutlin-3 or left untreated were co-cultured with *H. pylori* strain 7.13 for 6 hrs and analyzed for CagA protein phosphorylation using phospho-tyrosine antibody. CagA protein was used as a loading control.
